# Economic Boom and Busts Lead to Human Capital Changes? Evidence From Health Expenditure Changes in Emerging Economies

**DOI:** 10.3389/fpubh.2022.936004

**Published:** 2022-06-10

**Authors:** Yichi Zhang, Wei Deng, Ayesha Afzal, Ran Tao

**Affiliations:** ^1^Business School, Wuchang University of Technology, Wuhan, China; ^2^Lahore School of Economics, Lahore, Pakistan; ^3^Qingdao Municipal Center for Disease Control and Prevention, Qingdao, China

**Keywords:** economic stabilization, economic intervention, patient safety, health inequalities, business cycles, emerging national economies

## Abstract

This paper assesses data from 16 emerging economies between 2000-and 2020 to assess the relationship between business cycles and healthcare expenditure alongside other control variables. Using the Gaussian mixture model, this study analyses the relationship between healthcare spending and business cycles, urbanization, population age, environmental quality, and the gender ratio. The paper finds that there exists a counter-cyclical relationship between economic booms/recessions and healthcare expenditure such that spending decreases during booms and goes up during recessions. The study also finds evidence that environmental quality plays a vital role in influencing healthcare expenditure.

## Introduction

The COVID-19 pandemic arguably highlighted the gaps in healthcare provision around the world. Historically, there exists a strong relationship between healthcare expenditure and economic growth. As countries recover from the pandemic and resume activities as per normal, it will be interesting to see how the relationship between healthcare expenditure and business cycles has evolved (1). However, it is important to establish the trends that existed in the absence of the pandemic to determine the impact of future policies. Most experts anticipate that economics will continue to play a major role in the outlook for national health expenditures in emerging economies.

As a crucial part of human capital development, healthcare expenditure directly contributes to higher economic growth (2). It is also noted that countries with a higher proportion of resources allocated to healthcare tend to positively impact the overall health outcomes for the nation (3). The rationale for this relationship lies in the fact that when health expenditure is high, there is an improvement in the population's health in general, leading to greater human capital accumulation and improved productivity, and the results are economic booms (4, 5).

The issue has garnered much interest from academics, and there are multiple claims to support both sides of the argument. One school of thought argues that healthcare expenditure is procyclical, such that it moves with the business cycle (6). This argument hypothesizes that healthcare expenditure will increase in times of economic booms while declining during recessions (7). Some plausible arguments include the affordability of medical services and the impact on the environment (8, 9), which may lead to the deteriorating health of the populace (10).

Another conjecture is that the linkages between business cycles and healthcare expenditure depend on the income level in the economy, such that in low-income countries, the healthcare expenditure will behave in a counter-cyclical manner (11). It is also argued that health expenditure is also dependent on the overall health of the population and the population demographics (5, 12, 13).

This paper attempts to determine whether the health care expenditure is pro-cyclical or countercyclical in emerging economies (14). The paper moves beyond the five main emerging economies and includes economies making rapid strides to assess the impact of changing business cycles on healthcare expenditure along with a variety of control variables (15). For this analysis, the paper uses the Generalized Method of Moments (GMM) model to see how the healthcare expenditure in each of these economies changes in line with the business cycle. It also uses Granger-Causality tests to determine the direction of the relationship. The diverse sample also allows for insights into policy development for emerging economies with relatively underdeveloped health infrastructures (16). The larger sample of emerging economies (BRICS, CIVET, Next 11) used in this study for analyzing the relationship between business cycles and healthcare expenditures is a major reason for its ability to add to the existing literature (17). Furthermore, the study controls for population demographics like gender ratio, aging ratio, urbanization, and environmental quality to isolate the impact of economic booms and recessions.

The rest of the paper is organized as follows: Section Introduction presents the existing literature on Section Model concludes.

## Literature Review

A thorough assessment of the existing literature suggests that the impact of economic booms and busts on healthcare expenditure is still ambiguous and inconclusive. Experts argue that in periods of economic booms, people have higher disposable incomes, which improves their ability to cover medical expenses, which increases the overall expenditure on healthcare (6, 8–10). Furthermore, it has also been established that due to the rapid economic activity in periods of booms, the environmental quality deteriorates, which in turn takes a toll on public health, leading to increased expenditure on health (18–20). This view is also supported by research from (21, 22). However, Pu et al. (23) suggest that health expenditure keeps altering between counter cyclical and procyclical as the economy moves through changing business cycles.

In contrast, the literature suggests that this trend is reversed in low-income counties such that business cycles and healthcare expenditure are counter-cyclical (24). Rana et al. (11) argued that the impact of the business cycle on healthcare depends on the income level of pupation. Their research shows that people reduce their spending on healthcare during economic booms and increase it during recessions in low-income countries (25). This view is also supported by research from (26). Jakovljevic et al. (26) also argued that the aging population is one of the reasons for the differences between developed and emerging markets.

One existing theory also argues that the relationship between health expenditure and business cycles depends on the existing population health, as determined by population age, pre-existing conditions, gender division, and medical services provision (12, 27). Most of the evidence on the subject is drawn from developed countries (7, 21) with a limited focus on emerging economies. Bedir investigated the relationship in developing economies, recognizing that the economic and cultural differences in developed and developing economies also affect health expenditure behavior (28).

Lane (29), Talvi and Vegh (30), and Farooq and Ali (31) have confirmed that public healthcare expenditure follows the cyclicity of the business cycle, with an increase noticed in economic booms and a decrease during the recession—their research is limited to OECD and Latin American countries. Blöchliger and Égert (32) added that in countries with greater growth volatility and instability, this holds particularly true—recessions see a contraction of public healthcare expenditure, whereas it increases during booms (33). Cleeren et al. (34) argued that health expenditure follows the business cycle and trends of other shocks to the economy.

Shahri et al. (35) contended that all macroeconomic indicators impact on the overall healthcare expenditure. In the absence of controlling for their impact, the relationship between business cycles and health expenditure may be overstated, leading to the misallocation of resources to the health sector (36). Pakdaman et al. (3) build upon this further to add that the lack of transparency of national health accounts and delayed reporting of data further affect the efficacy of health expenditure policies and hinder its natural cyclicity (37).

## Data

This study utilizes data from 17 emerging economies. The companies form the following blocs: BRICS, CIVET, and Next 11—Table 1 illustrates.

**Table 1:** List of countries.

**Country**	**Bloc**	**Country**	**Bloc**	**Country**	**Bloc**
Brazil	BRICS	Columbia	CIVETS	Bangladesh	Next 11
Russia	BRICS	Indonesia	CIVETS	Iran	Next 11
India	BRICS	Vietnam	CIVETS	Mexico	Next 11
China	BRICS	Egypt	CIVETS	Nigeria	Next 11
South Africa	BRICS	Turkey	CIVETS	Pakistan	Next 11
		South Korea	CIVETS	Philippines	Next 11

Out of the 17 countries listed above, 16 have been used to construct the panel dataset for the years 2000-2020—South Korea has been omitted from calculations due to lack of healthcare data availability. The variables of interest are listed in Table 2:

**Table 2:** Description of variables.

**Type**	**Variable**	**Code**	**Source**
Dependent	Current health expenditure per capita (current US$)	HE1	World Development Indicators
	Current health expenditure (% of GDP)	HE2	World Development Indicators
Independent	Real GDP growth rate (%)	BC	IMF Economic Outlook Report
Control	Urban population (% of total population)	Urb	United Nations Population Division.
	Population ages 65 and above (% of the total population)	Age	United Nations Population Division.
	Population, male (% of the total population)	Gen	United Nations Population Division.
	CO2 emissions (metric tons per capita)	EQ	Climate Watch. 2020. GHG Emissions.

For the dependent variable, i.e., healthcare expenditure, we use current health expenditure per capita (HE1) and current health expenditure, % of GDP (HE2). The business cycle (BC) is estimated using the real GDP growth rate, which is in line with existing literature on the subject (6, 20, 38, 39). The control measure employed is the percentage of urban population (Urb), population aged above 65 (age), percentage of the population that is male (gender), and CO2 emissions per capita (EQ). These control measures are also supported by existing literature (38, 40–42).

The summary statistics for the data are in Table 3:

**Table 3:** Summary statistics.

	**Observations**	**Mean**	**Standard deviation**	**Min**	**Max**
BC	357	4.47	3.44	−9.60	14.60
HE1	320	245.19	240.44	8.36	1,031.58
HE2	320	4.86	1.86	1.85	9.59
Urb	357	55.04	18.18	23.59	87.07
Age	357	6.24	2.51	2.74	15.51
EQ	323	3.19	2.95	0.17	11.64
Gen	357	49.94	1.28	46.33	52.03

## Model

For the analysis of the panel dataset, we employ a generalized method of moments (GMM) framework to perform vector autoregression on the panel dataset (43). VAR in panel data settings was first introduced by Holtz-Eakin et al. (44) and quickly became a part of mainstream research methods. Under the assumption that errors are serially uncorrelated, the first differences model is consistent with the estimated equation by instrumenting lagged differences (45). We first perform the Levin-Lin-Chu unit-root test to check for stationarity in Table 4:

**Table 4:** Unit-root test.

	Adjusted t*	
BC	4.3961	Stationary
HE1	0.83	Stationary
HE2	−2.4537^**^	Non-stationary
Urb	−4.9078^***^	Non-stationary
Age	9.0411	Stationary
EQ	−0.7925	Stationary
Gen	−4.8195^***^	Non-stationary

*Source: Author's calculations*.

** *p < 0.05*,

*** *p < 0.1*.

Based on the data, we will next run two GMM first differences estimators, one for each dependent variable, followed by the Wald-test to check for Granger causality. The foremost reason for choosing the first differences estimator over the Arellano-Bond dynamic estimation is that our dataset comprises 16 countries across 20 years and is better suited to first differences.

## Results

The results from the GMM model are presented in Table 5:

**Table 5:** Robust GMM first differences panel modeling.

	**(1) HE1**	**(2) HE2**
L.HE1	1.055^***^ (0.053)	
L.HE2		0.686^***^ (0.06)
BC	−6.411^*^ (1.059)	−0.038^***^ (0.078)
Urb	1.591 (1.764)	−0.014 (0.013)
Age	0.827 (1.06)	0.069 (0.054)
EQ	20.62^*^ (9.74)	0.179^*^ (0.072)
Gen	−75.59^*^ (33.6)	0.056 (0.245)
Constant	−36.41^*^ (1,683.9)	−1.323^*^ (1.22)
*No. of countries*	16	16
Observations	256	256

*Standard errors in parenthesis*.

* *p < 0.01*,

*** *p < 0.1*.

The table shows that both measures for health expenditure (HE1 and HE2) are significantly related to their lagged value, such that the healthcare expenditure in a certain year has a significant positive impact on the expenditure for the subsequent year. We also see that healthcare expenditure has a significant negative relationship with the business cycle, suggesting that there is a counter-cyclical relationship between healthcare expenditure and the business cycle in emerging economies.

Next, we note that there exists a significant positive relationship between environmental quality (EQ) and healthcare expenditure, which is also supported by existing literature. During times of economic booms, greenhouse gas emissions tend to increase, thereby worsening the air quality. In turn, poor air quality leads to health issues and illnesses, prompting increased expenditure on healthcare.

The results also suggest that when using HE1 (per capita GDP), the gender ratio has a significant negative relationship with healthcare expenditure, suggesting that countries with a higher proportion of men in the economy tend to spend less on healthcare. However, we find no evidence of a relationship when using HE2 (healthcare expenditure as a percentage of GDP). We also do not find any evidence of a significant relationship between healthcare expenditure, age, and urbanization.

Table 6 presents the results from the Granger causality test for HE1, while Table 7 presents the results for HE2:

**Table 6:** Granger causality, HE1.

**Variables**	**chi^**2**^**	**Relationship**
HE1-BC	2.889	Unidirectional
BC-HE1	0.043^**^	
HE1-Urb	0.022	Unidirectional
Urb-HE1	2.244^*^	
HE1-Age	0.402	Unidirectional
Age-HE1	3.884^**^	
HE1-EQ	0.182	Unidirectional
EQ-HE1	0.002^*^	
HE1-Gen	0.04	No Granger Causality
Gen-HE1	1.026	

* *p < 0.01*,

** *p < 0.05*.

**Table 7:** Granger causality, HE2.

**Variables**	**chi^**2**^**	**Relationship**
HE2-BC	0.978	Unidirectional
BC-HE2	1.884^*^	
HE2-Urb	1.598	Unidirectional
Urb-HE2	4.463^**^	
HE2-Age	0.293	No Granger Causality
Age-HE2	0.495	
HE2-EQ	1.826	Unidirectional
EQ-HE2	2.656^*^	
HE2-Gen	1.917	No Granger Causality
Gen-HE2	0.304	

* *p < 0.01*,

** *p < 0.05*.

The results indicate that healthcare expenditure is a Granger caused by business cycles, suggesting that in times of boom and recession, they will cause a change in healthcare spending in emerging economies. Similarly, we find that urbanization Granger causes an increase in healthcare expenditure. As more people migrate to urban areas, they have easier access to healthcare facilities and therefore drive up healthcare expenditure. We also find that healthcare expenditure does not Granger cause any changes in urbanization. Similar to the GMM model, we also find that environmental quality Granger causes changes in health expenditure such that when carbon emissions per capita increase, they Granger cause an increase in health spending as the public health deteriorates.

On the whole, the results indicate that there exists a counter-cyclical relationship between business cycles and healthcare expenditure. In short, this means that the faster-emerging economies develop, the lower the healthcare expenditure will be (46). One possible explanation for this lies in the fact that during times of economic boom, there is an improvement in the overall living standards, therefore positively contributing to the health of the masses. As a result, there is a lesser need for medical services, which explains why economic booms are associated with lower health spending. This is also supported by (23). Our results suggest that the inverse relationship between business cycles and health expenditure lays the ground for policymakers to emphasize this aspect when devising growth and healthcare strategies for emerging economies.

As far as the control variables are concerned, we find evidence that environmental quality has a significant impact on health expenditure, such that the higher the carbon emissions per capita, the more people spend on healthcare due to adverse effects on wellbeing. We do not find evidence of a significant relationship with age, possibly because most countries in the sample have large proportions of youth (under 30). The paper also indicates a need to assess how changing population demographics and health affect the relationship between business cycles and health expenditure (47). It would also be interesting to see how the relationship between business cycles and health spending in emerging economies has changed following the COVID-19 pandemic and assess whether the shock to global health has influenced health spending behavior across countries and regions.

## Conclusion

This paper uses panel data from 16 countries over 20 years between 2000 and 2020 to assess the impact of business cycles and economic shocks on health expenditure in emerging economies. The selected countries have been derived from BRICS, CIVETS, and Next 11. The paper finds that there exists a significant negative relationship between economic booms and healthcare expenditure, indicating that as the standard of living improves during times of economic prosperity, there is an improvement in general health in emerging economies and therefore reduced spending on healthcare expenditure. The paper also finds evidence that there exists a significant relationship between worsening environmental quality and health spending, such that as carbon emissions increase, health-related issues are exacerbated, and therefore, health spending goes up. Our data does not indicate any relationship between health spending and the aging population in emerging economies.

### Policy Implications

Policymakers' most important implication is that while healthcare spending is reduced during economic booms, governments should be prepared for the increased need for medical resources during recessions and devise policies accordingly. A well-planned healthcare policy is even more important in the aftermath of the COVID-19 pandemic, given the number of emerging countries where the healthcare infrastructure was woefully inadequate to deal with the increased demand for resources. Moreover, when assessing green initiatives, the impact of environmental degradation on health outcomes needs to be considered to gauge the true impact of proposed policies.

## Data Availability Statement

Publicly available datasets were analyzed in this study. This data can be found here: https://data.worldbank.org/.

## Author Contributions

YZ and WD: conceptualization, software, data curation, and writing—original draft preparation. AA: methodology, writing—reviewing, and editing. RT: visualization and investigation. All authors contributed to the article and approved the submitted version.

## Conflict of Interest

The authors declare that the research was conducted in the absence of any commercial or financial relationships that could be construed as a potential conflict of interest.

## Publisher's Note

All claims expressed in this article are solely those of the authors and do not necessarily represent those of their affiliated organizations, or those of the publisher, the editors and the reviewers. Any product that may be evaluated in this article, or claim that may be made by its manufacturer, is not guaranteed or endorsed by the publisher.
